# Vaccination with an *in vitro* culture attenuated *Babesia bovis* strain safely protects highly susceptible adult cattle against acute bovine babesiosis

**DOI:** 10.3389/fimmu.2023.1219913

**Published:** 2023-07-31

**Authors:** Reginaldo G. Bastos, Janaina Capelli-Peixoto, Jacob M. Laughery, Carlos E. Suarez, Massaro W. Ueti

**Affiliations:** ^1^ Animal Disease Research Unit, United States Department of Agricultural - Agricultural Research Service, Pullman, WA, United States; ^2^ Department of Veterinary Microbiology and Pathology, College of Veterinary Medicine, Washington State University, Pullman, WA, United States

**Keywords:** bovine babesiosis, *Babesia bovis*, *in vitro* culture attenuated vaccines, *Babesia* immunity, *Babesia* vaccine

## Abstract

**Introduction:**

Live *in vivo* attenuated *Babesia bovis* vaccines produced by sequential passages in splenectomized calves have historically been used to control acute bovine babesiosis in endemic areas worldwide. However, several constraints prevent the widespread use of these vaccines, including the need for several splenectomized calves to produce vaccine batches, and potential inconsistent parasite attenuation, which contraindicates their use for highly *Babesia*-susceptible adult cattle. Thus, the use of vaccines based on well-defined *in vitro* culture attenuated *B. bovis* strains emerges as a more sustainable and efficient alternative. Previous work demonstrated that the culture attenuated strain Att-S74-T3Bo is non-tick transmissible and able to safely protect calves against needle challenge with a *B. bovis* virulent strain.

**Methods and results:**

Herein we evaluated safety and efficacy of Att-S74-T3Bo in preventing acute babesiosis in adult (>1.5 year of age) cattle. Results demonstrated that Att-S74-T3Bo vaccination of adult animals (n=5) induced self-limiting signs of acute infection and protected the vaccinated animals against challenge with the homologous virulent *B. bovis* strain Vir-S74-T3Bo. Att-S74-T3Bo-vaccinated adult cattle developed significant (P<0.05) monocytosis, with concomitant neutropenia and CD4^+^ leukopenia, in peripheral blood early after vaccination. Also, vaccinated animals developed a specific signature of pro- and anti-inflammatory cytokine expression in peripheral blood and significant levels of IgM, total IgG, IgG1, and IgG2 against the *B. bovis* immunodominant antigen RAP-1 CT. Strikingly, none of the vaccinated animals showed any signs of acute babesiosis after challenge with Vir-S74-T3Bo. In contrast, control adult cattle (n=5) showed pathognomonic symptoms of acute babesiosis, and significant decrease (P<0.05) in lymphocytes, monocytes, and neutrophils, starting on day 7 post-challenge. All control animals developed severe acute disease and were euthanized on days 10 through 12 days post-challenge.

**Discussion and conclusion:**

Evidence from this study indicates that Att-S74-T3Bo safely protects highly susceptible adult cattle against challenge with a homologous virulent strain of *B. bovis*. In conclusion, Att-S74-T3Bo may be considered as a potential efficient and sustainable attenuated candidate vaccine strain to control acute bovine babesiosis in highly susceptible adult cattle. Future studies should focus on increasing the number of animals vaccinated, duration of immunity, and efficacy of this attenuated strain against heterologous virulent parasite strains.

## 1 Introduction

Apicomplexan hemoparasites of the genus *Babesia* are the causative agents of babesiosis, a tickborne disease of vertebrates that has a detrimental impact on public and animal health (1, 2). Of particular concern is bovine babesiosis, also known as Texas cattle fever, considering the large number of cattle at-risk of developing the disease in tropical and sub-tropical areas of the world, and the devastating economic impact that it can have on animal protein production worldwide (2). The historical study performed by Smith and Kilbourne in the late 1800s not only defined bovine babesiosis as the first discovered arthropod-transmitted disease but also set the foundation for an intense and effective program to eradicate the *Babesia*-vector *Rhipicephalus microplus* ticks from the US and consequently eradication of the disease from the country in the early 1940s (3, 4). However, with current climate changes and consequential emergence and re-emergence of tick populations in the southern states of the US, along with the presence of exotic antelope species in the region and the large number of potential *Babesia*-infected cattle entering the US from Mexico, serious concerns have been raised regarding the re-introduction of bovine babesiosis into the country (5, 6). Should an outbreak of bovine babesiosis occur, the US cattle industry’s ability to produce animal protein would be significantly compromised with implication on food security.

*Babesia bovis* is the most virulent and one of the primary causative agents of bovine babesiosis; however, other *Babesia* species, such as *Babesia bigemina*, *Babesia divergens*, *Babesia ovata*, and *Babesia major*, have also been implicated in outbreaks among cattle herds worldwide (2, 7–9). Acute *B. bovis* infection in naïve animals is characterized by fever, anemia, drop in packed cell volume (PCV), anorexia, prostration, and high mortality. *B. bovis* acute infection can also result in neurological symptoms due to adhesion of infected red blood cells (iRBC) in the brain capillaries, a fatal condition that resembles severe cerebral malaria (10, 11). Animals that are able to survive the acute phase of the disease develop life-long chronic infection and become reservoirs for parasite acquisition and transmission by competent tick vectors. Currently available methods to minimize the impact of bovine babesiosis include the application of acaricides to control tick infestation, utilization of anti-*Babesia* drugs, and use of live *in vivo* attenuated vaccines. Unfortunately, none of these approaches are cost-effective and sustainable to control the disease in endemic areas and to prevent its introduction into at-risk regions. For instance, *in vivo* attenuated vaccines are relatively effective in protecting cattle against bovine babesiosis in endemic areas, but they present several constraints that preclude their wide-spread use. Among these limitations are: (a) the need of several splenectomized calves for vaccine preparation; (b) the potential risk of recombination between vaccine and field parasite strains during sexual reproduction inside tick vectors, which can result in the emergence of novel virulent daughter strains; and (c) insufficient parasite attenuation that prevents the vaccine use for highly *Babesia*-susceptible adult cattle (>1-year old) (12–14). Another limitation of the *in vivo* attenuated *Babesia* vaccines is the potential development of inadequate protection after vaccination, especially against heterologous parasite strains, which may cause outbreaks of acute bovine babesiosis in vaccinated herds of cattle (13, 15, 16). As a result of all these limitations, the use of live *in vivo* attenuated *B. bovis* vaccines is not currently licensed in the US. Pondering this scenario, and the current absence of subunit vaccines against *B. bovis*, or any other *Babesia* species, it becomes urgent to investigate effective and sustainable novel vaccines to prevent the devastating effects of acute bovine babesiosis.

Considering the need for developing control strategies against the disease, different factors, such as genetics, age, and immunological status, among others, are known to be implicated in determining the cattle’s susceptibility to acute *B. bovis* infection. It has been shown that European *Bos taurus* breeds are markedly more susceptible to the parasite than *Bos indicus* (2, 17). Also, it has been well-documented that young animals (<6 months of age) are more resistant to acute bovine babesiosis while adult cattle (>1 year of age) are significantly more susceptible (18–21). The causes for this age-related resistance against *Babesia* is not completely understood; however, it seems to be associated with early activation of innate immune mechanisms in calves rather than maternal immunity (18, 22). Interestingly, spleen removal results in recrudescence of the disease in cattle regardless of their age, and abrogation of the resistance in young animals, which indicates that splenic immunological mechanisms are involved in protection (23). In that context, the fact that live vaccines confer relatively solid protection and young cattle are resistant to *B. bovis* acute infection reveal that protective immunity can be achieved. Gaining more insight on the nature of the effective immunity mechanisms elicited in such protected animals may help develop efficient vaccines against bovine babesiosis.

It has been shown that long-term *in vitro* cultivation of *Babesia* can lead to attenuation, decreased population complexity, and selection of parasites that are no longer transmitted by competent ticks (24, 25). Considering that *Babesia* parasites are cultured in laboratories under standard protocols and controlled conditions using minimal animal products, the use of *in vitro* culture attenuated vaccine strains of *B. bovis* may emerge as a sustainable and more efficient alternative to *in vivo* attenuated strains for the control of acute bovine babesiosis. In a previous study, we demonstrated that infection with the *in vitro* culture attenuated *B. bovis* strain Att-S74-T3Bo safely protects young cattle against a lethal challenge with a homologous virulent strain, revealing novel immune correlates of protection against acute disease (26). In this work we expanded that knowledge and tested the hypotheses that: (a) Att-S74-T3Bo vaccination is safe for highly *Babesia*-susceptible adult cattle; and (b) that Att-S74-T3Bo-vaccinated animals are protected against challenge with a virulent homologous *B. bovis* strain.

## 2 Materials and methods

### 2.1 Parasite strains, vaccination, and challenge

The culture attenuated *B. bovis* strain Att- S74-T3Bo, originated from the parental T2Bo strain (27), was used for vaccination of adult cattle in this study. This strain has been continuously maintained in culture for more than 10 years, under standard conditions, as previously described (28). Recently, we demonstrated that Att-S74-T3Bo is not tick transmissible, has low population complexity compared to its parental virulent strain, and has an attenuated phenotype in calves (24, 25). For vaccination, a total of five male Holstein adult animals (>1.5-year of age) were inoculated intravenously (IV) with Att-S74-T3Bo iRBC (10^6^ iRBC/calf). Another group of five male Holstein cattle (>1.5-year of age) were inoculated IV with 10^6^ normal RBC (nRBC)/calf as controls. After Att-S74-T3Bo vaccination, all animals were monitored daily for clinical signs of acute babesiosis, including fever, drop in PCV, presence of parasites in peripheral blood, anorexia, prostration, and neurological signs. At 30 days after Att-S74-T3Bo inoculation, vaccinated and control animals were challenged IV with one 1-ml stabilate containing 10^7^ iRBC of the virulent homologous *B. bovis* strain Vir-S74-T3Bo. Previous studies have shown that a challenge dose of 10^7^ iRBC of Vir-S74-T3Bo induces severe acute disease in calves with the onset of clinical signs starting approximately 9-10 days after infection (24, 26, 29, 30). After challenge, all animals were monitored daily for clinical signs of acute bovine babesiosis, as described above. Average results of temperature and PCV at several timepoints after vaccination and challenge in all experimental animals were compared by a two-tailed *t* test using GraphPad Prism software version 9 (GraphPad Software, San Diego, CA). A P value <0.05 was considered statistically significant.

### 2.2 Parasite load in peripheral blood

Parasite load in peripheral blood of animals after vaccination and challenge was measured by real-time quantitative PCR (qPCR), as previously described (31). Briefly, blood from experimental animals was collected via jugular venipuncture into Vacutainer^®^ tubes containing ethylenediamine tetra acetic acid (EDTA) (BD Company, Franklin Lakes, NJ) at several timepoints after vaccination and challenge. Genomic DNA from whole blood was extracted using the QIAamp^®^ DNA Blood Mini Kit (QIAGen, Valencia, CA) following the manufacture’s protocol. DNA was used for qPCR to amplify the single copy *B. bovis msa-1* gene. For the *msa-1* qPCR, specific primers (5’ gatgcgtttgcacatgctaag 3’ and 5’ cgggtacttcggtgctctca 3’) and probe (FAM 5’-cacgctcaagtaggaaattttgttaaacctgga-3’ TAMRA) were used, and reactions were performed under the following conditions: 95°C for 10 min, 40 cycles of 95°C for 30 sec and 55.8°C for 15 sec, and extension at 72°C for 1 min. A *msa-1* standard curve was prepared with 10^0^ to 10^7^ plasmid copies, and all samples were run in technical triplicate, as described elsewhere (31). Results of parasite load in peripheral blood are presented as copies of *B. bovis msa-1* gene per 100 μl of blood. Average results of parasite load in vaccinated and control animals were compared by a two-tailed *t* test and a P value <0.05 was considered statistically significant.

### 2.3 Complete blood count

Complete cell blood count was evaluated in all experimental animals at several timepoints after vaccination and challenge using the ProCyte One™ Hematology Analyzer (IDEXX Laboratories, Westbrook, ME). Briefly, peripheral blood from the experimental animals was collected in Vacutainer^®^ tubes containing EDTA at several timepoints after vaccination and challenge. Blood samples were homogenized for 5 minutes, and the numbers of total leukocytes, lymphocytes, monocytes, neutrophils, and RBC were measured. Results of each leukocyte population are presented as 1,000 cells/μl of blood, and RBC are shown as 1,000,000 cells/μl of blood. Average results of cell count in all experimental animals were compared by a two-tailed *t* test, and a P value <0.05 was considered statistically significant.

### 2.4 Phenotypic analysis of peripheral blood mononuclear cells

Alterations in the phenotype percentage of peripheral blood mononuclear cells (PBMC) was examined by flow cytometric analysis, as previously described (26, 32). Peripheral blood from experimental animals was collected at several timepoints after infection in Vacutainer^®^ tubes containing EDTA. PBMC were isolated using Histopaque^®^-1077 (Sigma, St. Louis, MO), as per a standard protocol. Cells were then labeled with monoclonal antibodies to surface markers, followed by secondary antibodies (**Tables S1**, **S2**), using standard flow cytometry protocols, as described elsewhere (26, 32, 33). Flow cytometric analysis was performed using the Guava^®^ easyCyte™ flow cytometer (Luminex, Austin, TX), and data were acquired using the InCyte™ guavaSoft™ software version 3.1.1 (Luminex, Austin, TX). A single-color monoclonal antibody panel was used for phenotyping CD14^+^ monocytes, CD335^+^ NK cells, CD4^+^ T cells, CD8^+^ T cells, γδ T cells, and B cells in PBMC. A minimum of 20,000 events were collected for each cell population. After acquisition, data were analyzed using FCS Express™, version 6 (DeNovo™ Software, Pasadena, CA). Results are presented as percentage of target cell populations in PBMC. Average percentage of PBMC populations in experimental animals at several timepoints post-vaccination and -challenge were compared by a two-tailed *t* test. A P value <0.05 was considered statistically significant.

### 2.5 Humoral immune response

The presence of antibodies against the C-terminal segment of the *B. bovis* immunodominant antigen rhoptry associated protein 1 (RAP-1 CT) in serum from vaccinated and control animals was evaluated by indirect ELISA (iELISA), as previously described (34). Briefly, peripheral blood was collected in vacutainer with no anticoagulant, and serum samples were obtained by standard procedures. For iELISA, 96-well Immulon™ 2HB microtiter plates (Thermo Fisher Scientific, Waltham, MA) were coated overnight at 4°C with 50 µl of recombinant RAP-1 CT (2µg/ml). Plates were then washed three times using 200 µl blocking buffer (0.2% I-Block™ in 1xPBS with 0.1% Tween 20) and blocked with 300 µl of the same buffer for one hour at 30°C. After blocking, bovine serum samples were diluted 1/50 in blocking buffer, and 50 µl was added to triplicate individual wells. Plates were incubated for one hour at 30°C and then washed five times in 200 µl blocking buffer. After that, 50 µl of a 1/1000 dilution of anti-bovine IgM, total IgG, IgG1, or IgG2 peroxidase labeled secondary antibodies (SeraCare, Milford, MA) were added to each well, and plates were incubated for 45 minutes at 30°C. After incubation, plates were washed four times using 200 µl blocking buffer and two times with 200 µl 1xPBS with 0.1% Tween 20. Fifty-five µl of SureBlue™ TMB (SeraCare) was then added to each well and plates were incubated for 10 minutes. Reaction was stopped by adding 55 µl TMB stop solution (SeraCare), and absorbance was measured at 450 nm using an ELISA plate reader. Results are presented as normalized OD values, where the absorbance value at different time after vaccination and challenge of each individual animal was divided by the animal’s value obtained at day zero.

### 2.6 Cytokine ELISA

Concentration of bovine IL-1β, IL-4, IL-6, IL-8, IL-10, CXCL10, IFNγ, and TNFα in serum samples of vaccinated and challenged animals was evaluated by MILLIPLEX^®^ Bovine Cytokine/Chemokine Magnetic Bead (Millipore-Sigma, Burlington, MA) following the manufacture’s protocol. Minimal detectable levels were as follows: 0.1 pg/ml IL-1β, 12.8 pg/ml IL-4, 2.6 pg/ml IL-6, 2.2 pg/ml IL-8, 0.96 pg/ml IL-10, 0.6 pg/ml CXCL10, 0.1pg/ml IFNγ, and 12.8 pg/ml TNFα. Standard curves and quality controls for each cytokine in the multiplex assay were prepared following the manufacture’s protocol. The LX-200™ instrument (Luminex, Austin, TX) was used for the multiplex analysis. Data acquisition was done by xPONENT^®^ software (Luminex), and results were analyzed by Belysa^®^ software (Millipore-Sigma). Levels of cytokine concentration in peripheral blood of all experimental animals were compared by the Mann-Whitney test, and a P value <0.05 was considered statistically significant.

## 3 Results

### 3.1 Vaccination with *B. bovis* Att-S74-T3Bo induces self-limiting acute infection in adult cattle

**Figure 1:**
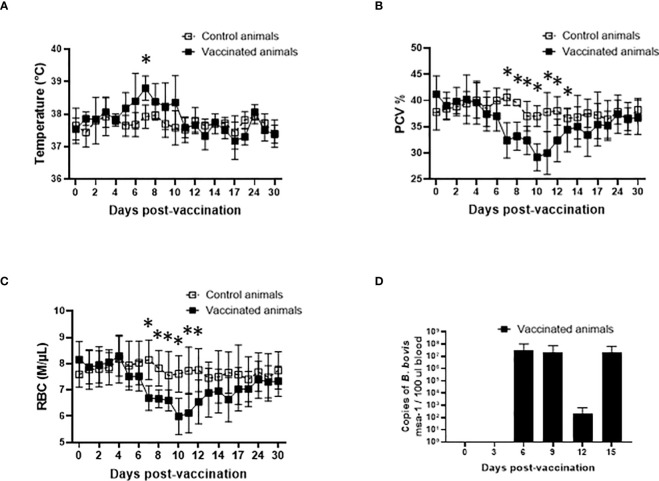
Temperature in Celsius degrees **(A)**, percentage of packed cell volume (PCV) **(B)**, absolute numbers of red blood cells (RBC), and parasite load in peripheral blood accessed by the copy numbers of the *B. bovis msa*-1 gene using real-time quantitative PCR **(D)** post-vaccination with the *in vitro* culture attenuated *B. bovis* strain Att-S74-T3Bo. Vaccinated animals (n=5). Control animals (n=5). * P<0.05.

One major impediment for the use of live *in vivo* attenuated *B. bovis* vaccines in endemic areas is that adult cattle may develop symptoms of acute disease and die due to vaccination. Consequently, the currently available live vaccines are not recommended for adult animals. Therefore, our first step in this study was to evaluate the safety of Att-S74-T3Bo for highly susceptible adult cattle. Results showed that animals that received 10^6^ iRBC of the attenuated parasite IV developed a mild but significant (P<0.05) increase of temperature at day 7 after vaccination compared to control non-vaccinated cattle (**Figure 1A**). Also, vaccinated animals presented a significant (P<0.05) drop in PCV compared to control animals from days 7 through 12 post-vaccination (**Figure 1B**). Increase of temperature and drop in PCV correlated with a significant (P<0.05) decrease of RBC in peripheral blood of vaccinated animals compared to controls from days 7 through 12 post-vaccination (**Figure 1C**). Data of parasite load in peripheral blood, determined by qPCR, indicated that Att-S74-T3Bo was able to infect and replicate in adult cattle. The first peak of parasite replication was observed at day 6 post-vaccination (**Figure 1D**), which preceded the increase of temperature and decrease of PCV, and consequent drop in RBC, on day 7 post-Att-S74-T3Bo inoculation. Importantly, all vaccinated animals recovered from the mild signs of acute infection without the administration of anti-pyretic treatment, anti-*Babesia* drug, or any additional veterinary intervention. Collectively, results demonstrated that Att-S74-T3Bo infected and caused self-limiting signs of acute disease with no evidence of neurological symptoms in the vaccinated animals. These features suggest that this *in vitro* culture attenuated *B. bovis* strain may be considered safe for administration into adult cattle.

### 3.2 Vaccination with *B. bovis* Att-S74-T3Bo protects adult cattle against challenge with homologous virulent parasite strain

**Figure 2:**
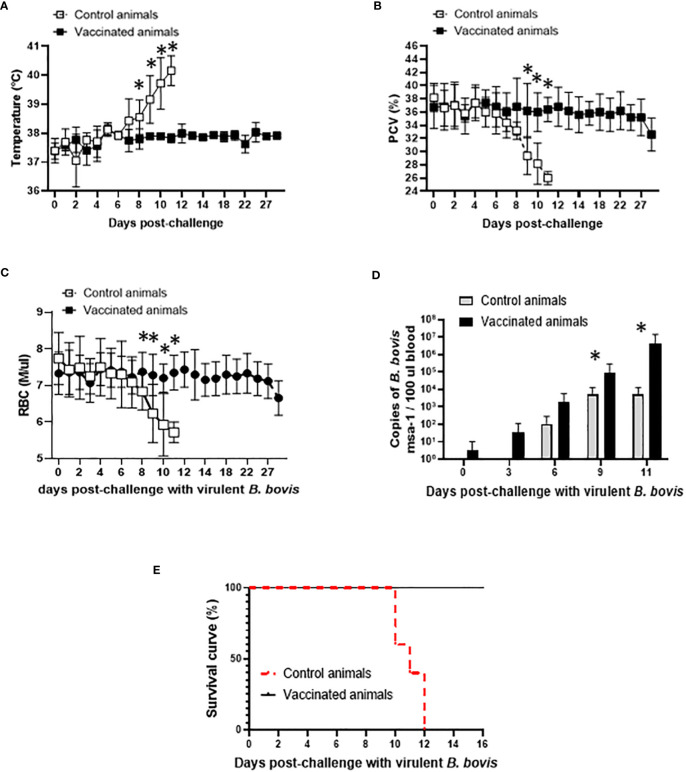
Temperature in Celsius degrees **(A)**, percentage of packed cell volume (PCV) **(B)**, absolute numbers of red blood cells (RBC) **(C)**, parasite load in peripheral blood accessed by the copy numbers of the *B. bovis msa*-1 gene using real-time quantitative PCR **(D)**, and Kaplan-Meier survival curve **(E)** after challenge with the virulent *B. bovis* strain Vir-S74-T3Bo. Vaccinated animals (n=5). Control animals (n=5). * P<0.05.

After demonstrating that Att-S74-T3Bo vaccination did not induce severe acute disease in the experimental adult cattle, we evaluated the development of protection in the vaccinated animals. Data indicated that Att-S74-T3Bo-vaccinated cattle survived challenge with the homologous virulent *B. bovis* strain Vir-S74-T3Bo (**Figure 2**). Vaccinated animals were monitored for 30 days and showed no significant alterations in temperature, PCV, and RBC in peripheral blood after Vir-S74-T3Bo challenge (**Figures 2A–C**). In contrast, all control animals developed pathognomonic signs of acute bovine babesiosis, including significant (P<0.05) increase of temperature and decrease of PCV and RBC starting at day 8 and 9 post-challenge, respectively (**Figures 2A–C**). In addition, control animals showed inappetence and lethargy, starting around day 8 post-challenge. Results of parasite load in peripheral blood indicated replication of *B. bovis* in both vaccinated and control animals after challenge (**Figure 2D**). Interestingly, levels of parasite in peripheral blood were significantly (P<0.05) higher in vaccinated cattle than in control animals on days 9 and 11 after challenge. Control cattle were humanely euthanized on days 10 (n=2), 11 (n=1), and 12 (n=2) post-challenge due to the severity of clinical signs of acute disease (**Figure 2E**). Altogether, these results demonstrated that highly susceptible adult cattle vaccinated with Att-S74-T3Bo are protected against challenge with a virulent homologous parasite strain.

### 3.3 Att-S74-T3Bo-vaccinated adult cattle developed a specific protective profile of leukocytes in peripheral blood

**Figure 3:**
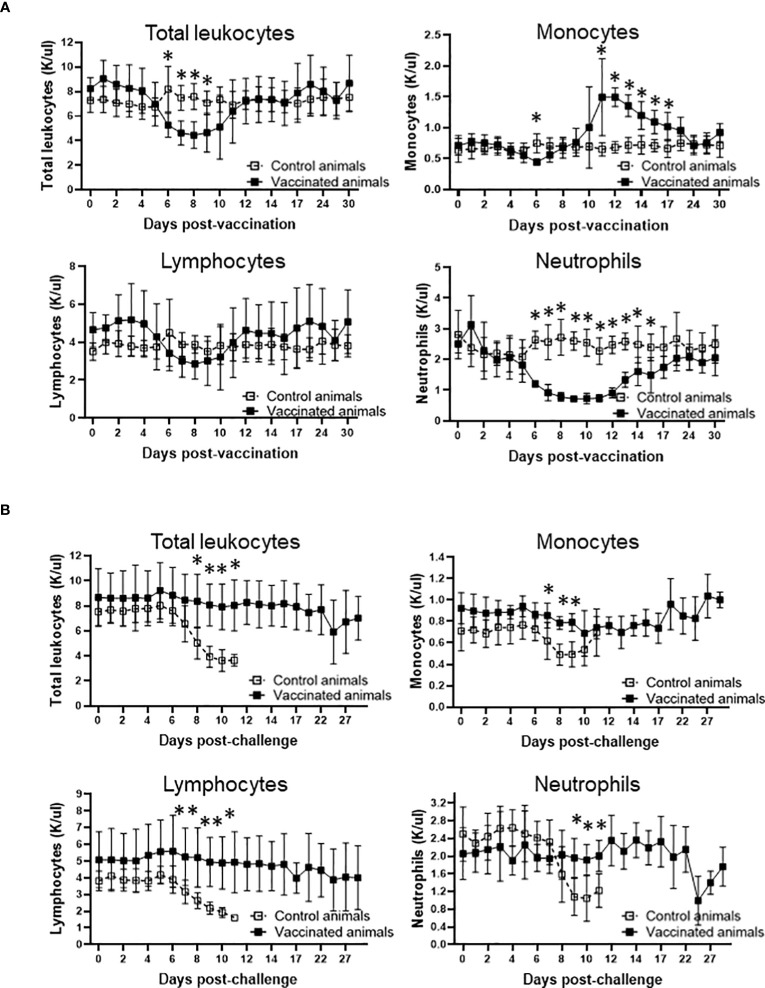
Profile of total leukocytes, lymphocytes, monocytes, and neutrophils in peripheral of vaccinated (n=5) and control (n=5) animals after vaccination with the *in vitro* culture attenuated *B. bovis* strain Att-S74-T3Bo **(A)** and challenge with the virulent *B. bovis* strain Vir-S74-T3Bo **(B)**. Results are presented as average of 1,000 cells per µl of blood (K/µl) of experimental animals. * P<0.05.

**Figure 4:**
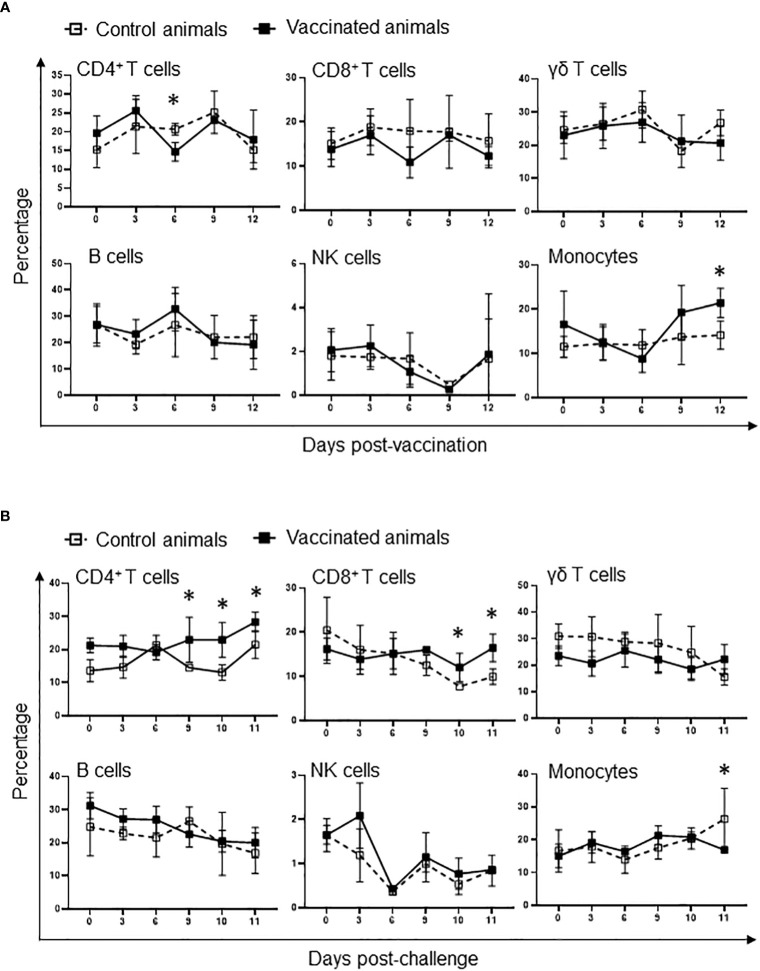
Flow cytometric analysis showing alterations of peripheral blood mononuclear cells (PBMC) after vaccination with the *in vitro* culture attenuated *B. bovis* strain Att-S74-T3Bo **(A)** and challenge with the virulent *B. bovis* strain Vir-S74-T3Bo **(B)**. Results are presented as average of percentage of 30,000 cells of experimental animals. Vaccinated animals (n=5). Control animals (n=5). * P<0.05.

The evidence that Att-S74-T3Bo vaccination protected adult cattle against challenge with Vir-S74-T3Bo led us to investigate alterations of leukocytes in peripheral blood from vaccinated protected animals in comparison to controls. As a first step in this investigation, peripheral blood of experimental animals was evaluated using a hematology analyzer to determine the alterations in the absolute numbers of total leukocytes, lymphocytes, monocytes, and neutrophils at different timepoints after vaccination and challenge (**Figure 3**). Results showed a significant (P<0.05) decrease of total leukocytes in Att-S74-T3Bo-vaccinated cattle on days 6 to 9 post-vaccination (**Figure 3A**). Even though no significant changes were observed in the levels of lymphocytes, vaccinated cattle showed a tendency toward lymphocytopenia between days 6 and 9 post-vaccination compared to controls (**Figure 3A**). In addition, Att-S74-T3Bo-vaccinated cattle showed a marked decrease (P<0.05) of neutrophils in peripheral blood from days 6 to 15 post-vaccination (**Figure 3A**). Interestingly, vaccinated animals developed a significant (P<0.05) monocytopenia at day 6 followed by marked monocytosis in peripheral blood, compared to controls, from day 11 to 17 post-vaccination (**Figure 3A**). Control animals showed significant (P<0.05) decrease of total leukocytes, lymphocytes, monocytes, and neutrophils in peripheral blood starting around day 7 to 8 post-challenge (**Figure 3B**). In contrast, no significant alterations in the absolute numbers of total leukocytes, lymphocytes, monocytes, and neutrophils were observed in peripheral blood of Att-S74-T3Bo-vaccinated cattle after challenge (**Figure 3B**). Next, we examined the profile of PBMC in experimental cattle after Att-S74-T3Bo vaccination and Vir-S74-T3Bo challenge (**Figure 4**). Gate strategy for the flow cytometric analysis to determine the percentage of CD14^+^ monocytes, CD335^+^ NK cells, CD4^+^ T cells, CD8^+^ T cells, γδ T cells, and B cells in PBMC of experimental animals is shown in the **Supplemental Figure 1** (**Figure S1**). Results demonstrated a significant (P<0.05) decrease of CD4^+^ T cells in peripheral blood of cattle at day 6 post-vaccination compared to unvaccinated control animals (**Figure 4A**). Also, data demonstrated a significant (P<0.05) increase of the percentage of monocytes in peripheral of vaccinated cattle starting at day 12 post-Att-S74-T3Bo inoculation compared to controls (**Figure 4A**), confirming the results obtained from the whole blood analysis (**Figure 3A**). In addition, vaccinated animals presented a tendency toward decrease of CD8^+^ T cells in peripheral blood compared to controls at day 6 post-vaccination; however, no significant differences were observed (**Figure 4A**). No significant differences in the percentage of γδ T cells, NK cells, and B cells were observed in peripheral blood of vaccinated cattle in comparison to controls (**Figure 4A**). The percentage of CD4^+^ and CD8^+^ T cells decreased significantly (P<0.05) in peripheral blood of control animals, compared to vaccinated cattle, starting at days 9 and 10 post-Vir-S74-T3Bo challenge, respectively (**Figure 4B**). A significant (P<0.05) monocytosis in PBMC of control animals compared to vaccinated cattle was observed at day 11 post-challenge, by that time two control animals had to be euthanized due to the severity of the disease while the other three remaining cattle were showing severe signs of acute babesiosis. No significant differences in the percentage of γδ T cells, NK cells, and B cells in peripheral blood of vaccinated cattle compared to controls were observed after Vir-S74-T3Bo challenge (**Figure 4B**). Together, the results demonstrate the development of a specific pattern of alterations in leukocytes in peripheral blood of protected adult cattle vaccinated with Att-S74-T3Bo, which was mainly characterized by increase of monocytes with concomitant decrease of CD4^+^ T cells early after Att-S74-T3Bo vaccination.

### 3.4 Protected Att-S74-T3Bo-vaccinated cattle developed early expression of pro- and anti-inflammatory cytokines in peripheral blood with significant humoral response to *B. bovis* RAP-1 CT

**Figure 5:**
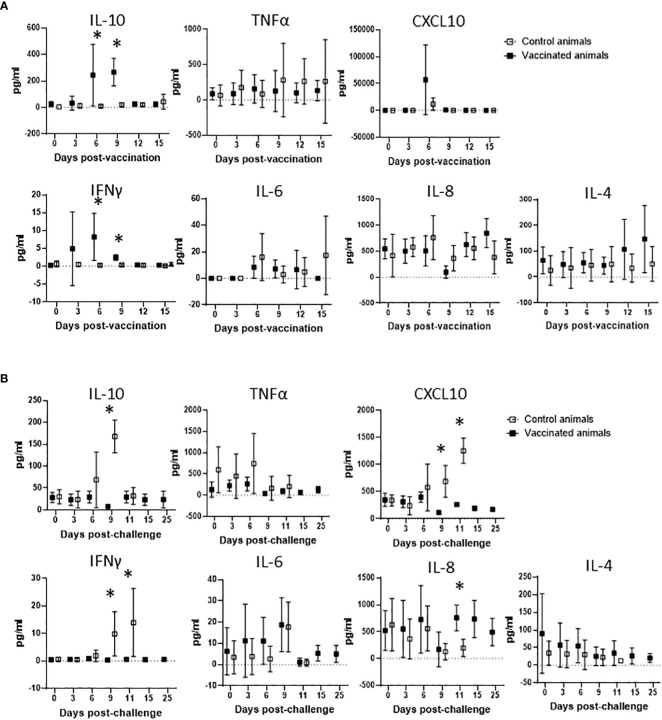
Expression profile of IL-10, TNFα, CXCL10, IFNγ, IL-6, IL-8, and IL-4 in peripheral blood of cattle vaccinated with the *in vitro* culture attenuated *B. bovis* strain Att-S74-T3Bo **(A)** following challenge with the virulent *B. bovis* strain Att-S74-T3Bo **(B)**. Cytokine results are presented as means of picograms per ml of blood (pg/ml) of experimental animals. Vaccinate animals (n=5). Control animals (n=5). * P<0.05.

**Figure 6:**
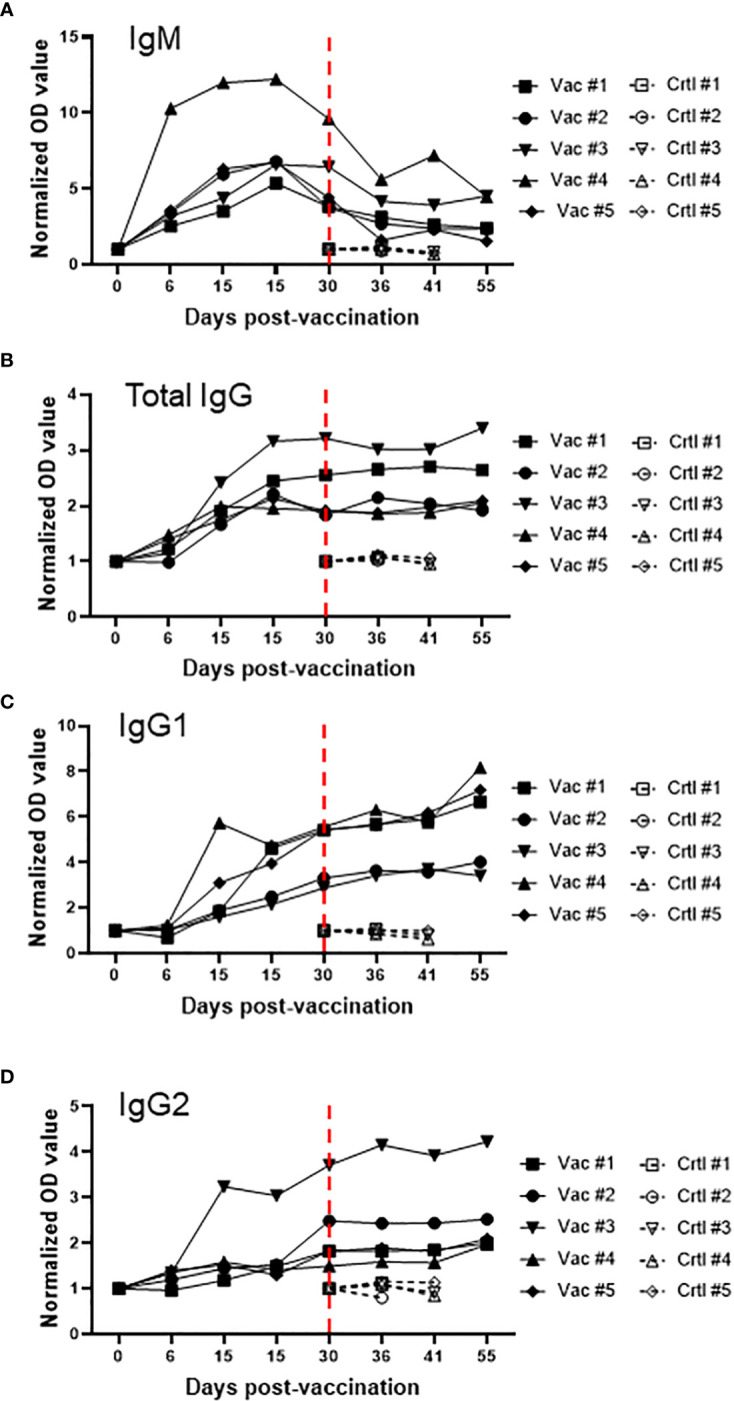
Levels of IgM **(A)**, total IgG **(B)**, IgG1 **(C)**, and IgG2 **(D)** against the immunodominant antigen rhoptry associated protein 1 C-terminal (RAP-1 CT) after vaccination with the *in vitro* culture attenuated *B. bovis* strain Att-S74-T3Bo and challenge with the virulent *B. bovis* strain Vir-S74-T3Bo. Results are presented as normalized optical density of individual experimental animals. Animals vaccinated with Att-S74-T3Bo and challenged with Vir-S74-T3Bo (Vac). Control animals challenged with Vir-S74-T3Bo (Crtl). Dashed red lines indicate day zero of Vir-S74-T3Bo challenge.

After demonstrating that Att-S74-T3Bo vaccinated adult cattle are protected against challenge with Vir-S74-T3Bo and that protected animals developed a signature pattern of leukocytes in peripheral blood, next we examined the immune responses of the experimental animals (**Figure 5**). We observed a significant increase in the level of IL-10 in peripheral blood of vaccinated cattle between days 6 and 9 post-Att-S74-T3Bo inoculation (**Figure 5A**). Similarly, IFNγ was significantly elevated in peripheral blood of vaccinated animals on days 6 and 9 post-vaccination (**Figure 5A**). In addition, no significant differences were detected in the levels of CXCL10, TNFα, IL-6, IL-8, and IL-4 after vaccination (**Figure 5A**). Control cattle showed significant (P<0.05) increase in CXCL10, IFNγ, and IL-10 at day 9 post-challenge, compared to Att-S74-T3Bo-vaccinated animals. In addtion, a significant increase in the concentration of IL-8 was observed in vaccinated animals at day 11 post-challenge (**Figure 5B**). No significant differences were detected in the level of TNFα, IL-6, and IL-4 in vaccinated and control animals after challenge (**Figure 5B**). All Att-S74-T3Bo-vaccinated cattle developed detectable levels of IgM against the immunodominant antigen *B. bovis* RAP-1 CT as early as 6 days post-inoculation (**Figure 6A**). Also, marked levels of IgG, IgG1, and IgG2 against RAP-1 CT were detected, starting at day 15 post-inoculation, in all five Att-S74-T3Bo-vaccinated animals (**Figures 6B–D**). Importantly, no boost effect was observed in the levels of IgM and IgG after challenging the vaccinated animals with the virulent *B. bovis* strain. Noteworthy, no levels of detectable IgM and IgG against RAP-1 CT were observed in the control animals that succumbed to acute disease on days 10 through 12 post-challenge (**Figure 6**). In summary, data showed that vaccinated, protected adult cattle developed a balance expression profile of pro- and anti-inflammatory cytokines in peripheral blood, as demonstrated by the levels of IL-10, CXCL10 and, IFNγ after Att-S74-T3Bo vaccination and challenge. Also, Att-S74-T3Bo vaccination induced markedly levels of antibodies that may play a role in protecting the animals from challenge with the virulent parasite strain. In addition, data showed that control animals developed a late cytokine response compared to cattle inoculated with attenuated parasites.

## 4 Discussion

In this study we demonstrate that vaccination with the live *in vitro* culture attenuated *B. bovis* strain Att-S74-T3Bo is safe and protects adult cattle against acute bovine babesiosis. We also identified a pattern of leukocytes and cytokine expression in peripheral blood of protected animals, which increments the knowledge on correlates of protection against *B. bovis* acute infection found in a previous study (26). It has been well-documented that live *in vivo* attenuated *Babesia* vaccines generally protects cattle against acute bovine babesiosis (13). Despite this fact, these vaccines present several constraints that prevent their wide-spread use in cattle, especially in areas that are non-endemic for *Babesia* parasites, including the US. Among these limitations, at least two are a major concern. One is the need of a large number of animals for vaccine production, and the other is the risk of vaccinated cattle, especially adult animals (>1-year old), to develop acute disease due to vaccination, which prevents its use for highly susceptible adult cattle. In this study we started addressing these two concerns by using Att-S74-T3Bo, a previously defined *in vitro* culture attenuated strain of *B. bovis* (24–26). Despite of the reduced number of animals used in the present study, the data support the hypotheses that vaccination with Att-S74-T3Bo is safe for highly susceptible adult cattle and that it induces protection against challenge with a virulent homologous *B. bovis* strain. Results demonstrate that Att-S74-T3Bo-vaccinated cattle developed mild self-limiting acute infection, and that vaccination elicited a protective immune response against challenge with Vir-S74-T3Bo.

Currently available methodology to produce live *in vivo* attenuated *B. bovis* vaccines was developed in Australia more than 100 years ago (21). After that, this approach has been refined and slightly modified but in essence it consists of sequentially infecting several splenectomized calves with virulent *B. bovis* parasites. After approximately 20 cycles of infection/passages in individual splenectomized naïve calves, attenuate parasites are selected from the original population. Even though the genotypic and phenotypic differences between virulent and attenuated parasite populations after the passages in splenectomized calves remain unknown, the emerging attenuated parasites are then used as a live blood-based vaccine (2, 15, 16, 21). In this context, vaccination of cattle with live *in vivo* attenuated *B. bovis* parasites has been historically used as an approach to control acute babesiosis in endemic areas (35). However, field studies have reported failures of these vaccines with the emergence of outbreaks and development of acute disease in vaccinated herds of cattle. Outbreaks in vaccinated animals are associated with the presence of field isolates that are able to break through the vaccine immunity (36). On the other hand, attenuation through passages in splenectomized calves is both a biologically and logistically challenging process, and inadequate attenuation of vaccine strains is not uncommon. As a result, development of acute disease caused by vaccination may happen due to deficient selection of attenuated parasite populations during the passages in splenectomized calves or other factors, such as the breed and immunological status of the vaccinated animals, and the presence in the field of heterologous and/or homologous highly virulent strains. This is of particular concern considering the use of *in vivo* attenuated *B. bovis* vaccines for highly susceptible adult cattle. Consequently, these vaccines are only recommended for young (< 6 months of age) and not to adult animals (35). Another important point to be considered for the use of *in vivo* attenuated *B. bovis* vaccines is the possibility of recombination between vaccine and field strains of the parasite during sexual reproduction inside the tick vectors. Such situation could potentially produce new virulent parasite strains that may escape the vaccine immunity. Relevance of the present study relies on the fact of using the previously defined *in vitro* culture attenuated *B. bovis* strain Att-S74-T3Bo that safely protects highly susceptible adult cattle against challenge with a virulent homologous *B. bovis* strain. Collectively, this information adds to previous work showing that Att-S74-T3Bo has less population complexity compared to its parental strain and that it is not tick-transmissible (24, 25). Another major downside facing the wide-spread use of *in vivo* attenuated *B. bovis* vaccines is the difficulties in standardizing and large-scale producing parasite vaccine strains using splenectomized calves (16, 35). To address these concerns, alternative methods to attenuate *Babesia* parasites have been tested, including the use of chemical, radiation, and passages in *in vitro* culture (37–40). Here we show that a dose of 10^6^ *in vitro* culture attenuated parasites is enough to safely protect adult cattle from acute disease caused by a homologous virulent *B. bovis* strain. Considering that the volume of *B. bovis* cultures can be easily expanded using standard laboratory protocols, promoting the increase in the percentage of parasitized erythrocytes, which can reach approximately 40%, it is predicted that no major issues are expected regarding standardization and large-scale production of this *in vitro* attenuated vaccine strain. Altogether, results presented by this study represent an important advance regarding the development of a sustainable, safe, and efficient live *in vitro* attenuated vaccine to control acute bovine babesiosis in adult cattle.

It was previously shown that Att-S74-T3Bo inoculation protects calves (<1 year of age) against challenge with a homologous virulent strain (26). Protected calves showed increase of monocytes with concomitant decrease of neutrophils and CD4^+^ T lymphocytes in peripheral blood on day 3 to 7 post-Att-S74-T3Bo inoculation. Here we demonstrated that Att-S74-T3Bo is also effective in protecting adult cattle and that the protected animals showed a similar pattern of PBMC alteration compared to young animals. However, in this study, the adult protected cattle developed a delayed monocytosis, neutropenia and CD4^+^ lymphopenia in peripheral blood compared to the previous observation in young animals (26). Interestingly, here we observed monocytopenia and the absence of monocytosis in control animals, which correlates with the onset of clinical signs of acute disease. Taken together, we infer that increased number of monocytes in peripheral blood after vaccination is a *sine qua non* event to elicit protection against acute *B. bovis* infection regardless the cattle age. Of note, it has also been shown that recruitment and activation of monocytes and macrophages are critical to control parasitemia of lethal and non-lethal *Plasmodium* infection (41, 42), which is consistent with the findings of this study. Monocytes have been recently categorized into subsets based on the expression of the surface markers CD14 and CD16 (32, 43, 44). Therefore, future studies are needed to investigate the kinetics of monocyte subsets in peripheral blood of protected cattle after Att-S74-T3Bo vaccination in comparison with control animals. Additional investigation is also necessary to determine the profile of cytokine expression in the monocyte subsets in protected cattle.

It has been demonstrated that development of a protective immune response against *B. bovis* involves a fine balance of early activation of innate immune mechanisms, and subsequent elicitation of adaptive humoral and cellular responses (26, 45, 46). Interestingly, the association between protection and development of a balance of pro-inflammatory and regulatory cytokines was also demonstrated in *Plasmodium* spp. and *Theileria* spp (47–49). Findings of this current study agree with this assumption by demonstrating that vaccinated protected adult animals showed early systemic expression of IL-10, a regulatory anti-inflammatory cytokine, and concomitant elevation of IFNγ, a pro-inflammatory cytokine. In contrast, control animals presented similar but delayed pattern of cytokines following challenge with a virulent *B. bovis* strain. Considering the elevated levels of IFNγ and CXCL10 in controls animals on day 9 post-challenge, we speculate that this untimely systemic production of pro-inflammatory cytokines may account for the severity of acute disease caused by the virulent strain in adult animals, as previously mentioned (18). It is also possible to consider that control animals experienced a transient immunosuppression with subsequent exacerbation of the inflammatory immune response after challenge, which may be in part responsible for the clinical signs of acute bovine babesiosis and death of adult animals. In this study, Att-S74-T3Bo-vaccinated cattle developed significant and early levels of IgM and IgG against the *B. bovis* immunodominant antigen RAP-1 CT, which is in agreement with previous observation (26). In contrast, control animals succumbed to acute disease around days 10 to 12 post-Vir-S74-T3Bo challenge without detectable levels of anti-RAP-1 CT antibodies. Therefore, it is plausible to assume that humoral immune response against *B. bovis* antigens may play a role in protecting the vaccinated animals after challenge. Collectively, results of blood cell alterations, cytokine expression in peripheral blood, and humoral immune response in vaccinated protected adult cattle increment the information on correlates of immune protection against *B. bovis* and may be relevant for vaccine development.

In conclusion, here we demonstrate that, under the present experimental conditions, Att-S74-T3Bo vaccination is safe for adult cattle and that vaccinated animals are protected against challenge with the homologous virulent strain Vir-S74-T3Bo. By using a sensitive method for cytokine detection and a systemic approach to investigate alterations of leukocytes in peripheral blood after vaccination and challenge, we identified markers for protection and disease progression in highly susceptible adult cattle. Considering this and previous studies, we are building a solid body of information on the Att-S74-T3Bo strain (24–26, 50). It is now known that this attenuated long-term cultured *B. bovis* strain presents a low population complexity compared to its parental strain (24), it is not tick-transmissible (25), and it protects calves against challenge with homologous virulent strain (26). Results from this study increment previous knowledge and provide additional evidence on the safety and efficacy of Att-S74-T3Bo in protecting adult cattle against acute bovine babesiosis. Future studies should determine the duration of the immunity induced by Att-S74-T3Bo vaccination and its efficacy against heterologous virulent strains of *B. bovis* and challenge with *B. bovis*-infected ticks.

## Data availability statement

The original contributions presented in the study are included in the article/**Supplementary Material**. Further inquiries can be directed to the corresponding author.

## Ethics statement

The animal study was reviewed and approved by Washington State University Institutional Animal Care and Use Committee.

## Author contributions

Conception and design: RB, CS, MU. Collection and assembly of data: RB, JC-P, JL. Data analysis and interpretation: RB, JC-P, JL, CS, MU. Manuscript writing: All authors. Final approval of manuscript: All authors.
