# Supplementary figures and images for: Vaccination with an in vitro culture attenuated Babesia bovis strain safely protects highly susceptible adult cattle against acute bovine babesiosis
Source: Front Immunol. 2023 Jul 31;14:1219913. doi: 10.3389/fimmu.2023.1219913 (PMC10424928; doi:10.3389/fimmu.2023.1219913)

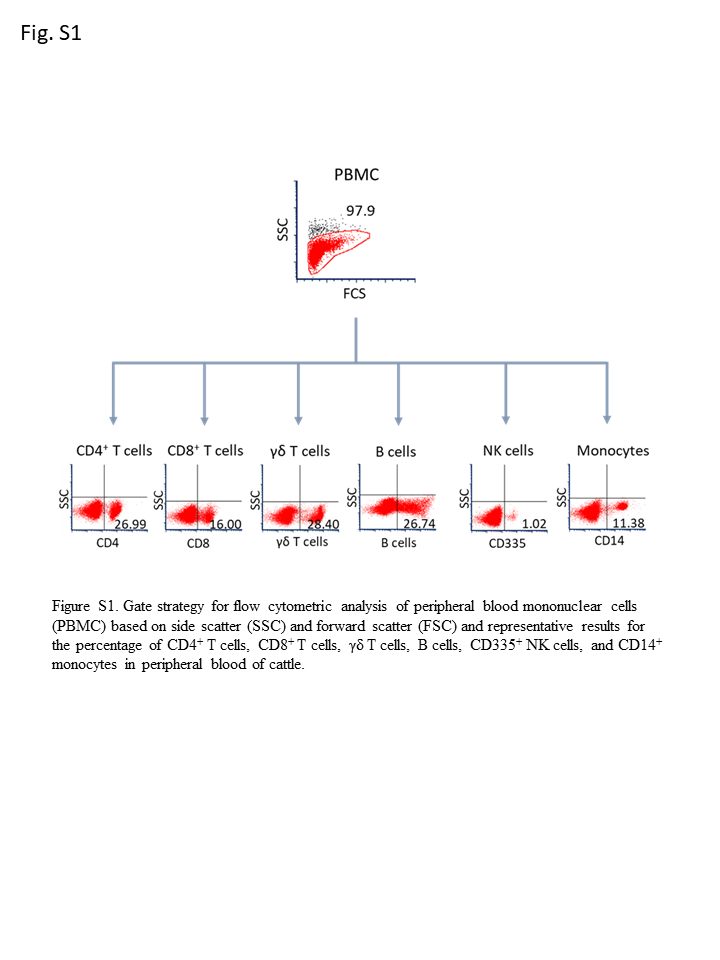

Supplement: Supplementary file 1 [file Image_1.tif]
