# Supplementary material for: Vaccination with an in vitro culture attenuated Babesia bovis strain safely protects highly susceptible adult cattle against acute bovine babesiosis
Source: Front Immunol. 2023 Jul 31;14:1219913. doi: 10.3389/fimmu.2023.1219913 (PMC10424928; doi:10.3389/fimmu.2023.1219913)
Supplement: Supplementary file 2 [file Table_1.docx]

Table S1. List of monoclonal antibodies used in this study to investigate the percentage monocytes, NK cells, T cells, γδ T cells, and B cells in peripheral blood mononuclear cells.

| Antibody | Isotype | Specificity | Source |
| --- | --- | --- | --- |
| CAM36A | IgG1 | CD14 monocytes | WSU Monoclonal Center |
| AKS1 | IgG1 | CD335 NK cells | Bio-Rad |
| ILA11A | Ig2a | CD4 T cells | WSU Monoclonal Center |
| 7C2B | Ig2a | CD8 T cells | WSU Monoclonal Center |
| GB21A | IgG2b | γδ T cells | WSU Monoclonal Center |
| BAQ155A | IgG1 | B cells | WSU Monoclonal Center |

Table S2. List of secondary reagents used in this study for flow cytometric analysis.

| Antibody Identification | Source |
| --- | --- |
| Alexa Flour™ 488 goat anti-mouse IgG1 | Thermo Fisher Scientific |
| Alexa Flour™ 647 goat anti-mouse IgG1 | Thermo Fisher Scientific |
| Alexa Flour™ 647 goat anti-mouse IgG2a | Thermo Fisher Scientific |
| Alexa Flour™ 647 goat anti-mouse IgG2b | Thermo Fisher Scientific |
| Alexa Flour™ 647 goat anti-mouse IgG | Thermo Fisher Scientific |
